# DNA replication arrest leads to enhanced homologous recombination and cell death in meristems of rice OsRecQl4 mutants

**DOI:** 10.1186/1471-2229-13-62

**Published:** 2013-04-12

**Authors:** Yong-Ik Kwon, Kiyomi Abe, Masaki Endo, Keishi Osakabe, Namie Ohtsuki, Ayako Nishizawa-Yokoi, Akemi Tagiri, Hiroaki Saika, Seiichi Toki

**Affiliations:** 1 Plant Genome Engineering Research Unit, National Institute of Agrobiological Sciences, 2-1-2 Kannondai, Tsukuba, Ibaraki, 305-8602, Japan; 2 Functional Plant Research Unit, National Institute of Agrobiological Sciences, 2-1-2 Kannondai, Tsukuba, Ibaraki, 305-8602, Japan; 3 Graduate School of Science and Engineering, Saitama University, 255 Shimo-okubo, Sakura, Saitama, 338-8570, Japan; 4 Graduate School of Nanobioscience, Yokohama City University, 22-2, Seto, Kanazawa, Yokohama, 236-0027, Japan; 5 Kihara Institute for Biological Research, Yokohama City University, 641-12 Maioka, Yokohama, 244-0813, Japan

**Keywords:** Rice, RecQ helicase, OsRecQl4, DNA replication, DNA double-strand break, Homologous recombination, Meristem

## Abstract

**Background:**

Mammalian BLM helicase is involved in DNA replication, DNA repair and homologous recombination (HR). These DNA transactions are associated tightly with cell division and are important for maintaining genome stability. However, unlike in mammals, cell division in higher plants is restricted mainly to the meristem, thus genome maintenance at the meristem is critical. The counterpart of *BLM* in *Arabidopsis* (*AtRecQ4A*) has been identified and its role in HR and in the response to DNA damage has been confirmed. However, the function of *AtRecQ4A* in the meristem during replication stress has not yet been well elucidated.

**Results:**

We isolated the *BLM* counterpart gene *OsRecQl4* from rice and analyzed its function using a reverse genetics approach. *Osrecql4* mutant plants showed hypersensitivity to DNA damaging agents and enhanced frequency of HR compared to wild-type (WT) plants. We further analyzed the effect of aphidicolin—an inhibitor of S-phase progression via its inhibitory effect on DNA polymerases—on genome stability in the root meristem in *osrecql4* mutant plants and corresponding WT plants. The following effects were observed upon aphidicolin treatment: a) comet assay showed induction of DNA double-strand breaks (DSBs) in mutant plants, b) TUNEL assay showed enhanced DNA breaks at the root meristem in mutant plants, c) a recombination reporter showed enhanced HR frequency in mutant calli, d) propidium iodide (PI) staining of root tips revealed an increased incidence of cell death in the meristem of mutant plants.

**Conclusions:**

These results demonstrate that the aphidicolin-sensitive phenotype of *osrecql4* mutants was in part due to induced DSBs and cell death, and that OsRecQl4 plays an important role as a caretaker, maintaining genome stability during DNA replication stress in the rice meristem.

## Background

DNA damage is thought to be induced not only by exogenous genotoxic stresses such as UV and ionizing radiation but also by intrinsic endogeneous stresses such as DNA replication errors and the oxidative stress associated with cell metabolism. Unlike animals, plants cannot escape from exogenous genotoxic stresses. Furthermore, in plants, DNA replication sites are restricted mainly to meristematic tissues, and differentiation of the germ line is not predetermined; plants thus need an efficient and flexible genome maintenance system. However, very little is known about the mechanism maintaining genome stability in response to DNA replication stresses.

RecQ helicases are members of the ATP-dependent helicase family and play important roles in maintaining genome stability in eukaryotic cells during DNA repair [1,2], DNA replication [3,4], telomere maintenance [5], and homologous recombination (HR) processes [6,7]. Yeast has a single RecQ gene, *Sgs1*, but mammals and plants have multiple RecQ genes, humans having five and Arabidopsis seven [8]. Interestingly, overlapping and distinct roles of RecQ proteins have been reported in humans [6,9]. For example, defects in *BLM*, *WRN* and *HsRecQ4* are found in Bloom’s, Werner, and Rothmund-Thomson syndromes, respectively [10-12]. Patients with these syndromes exhibit genomic instability and are predisposed to cancer, and their cells are hypersensitive to DNA-damaging agents. During DNA replication and maintenance of the telomere, the role of BLM overlaps with that of WRN. However, BLM promotes exonuclease 1-mediated DNA resection during the initial step of DSB repair, whereas other RecQ helicases do not promote this step [13,14]. Strikingly, patients with the *BLM* defect show enhanced sister chromatid exchange (SCE) frequencies [15], indicating that BLM suppresses HR in the course of DNA replication. Moreover, BLM interacts with replication protein A (RPA) and topoisomerase III [16,17]. It has been revealed recently that BLM plays a role in recovery from DNA replication arrest [18,19]. Therefore, BLM is likely to be a multi-functional protein involved in several aspects of genome maintenance in mammals. Many studies to date have focused on DNA replication maintenance, given the known association between HR and DNA replication [20-22].

Two *BLM* homologs, *AtRecQ4A* and *AtRecQ4B*, have been isolated in *Arabidopsis* and their functions analyzed using mutant plants [23,24]. *atrecq4A* mutants showed enhanced frequency of HR during normal growth condition and hypersensitivity to DNA damaging agents compared to wild-type (WT) plants. In contrast, *atrecq4B* mutants had a reduced frequency of HR and did not show sensitivity to DNA-damaging agents compared to WT plants despite the high degree of identity of the two RecQ4s [24]. Recently, it was reported that AtRecQ4A is required for efficient synthesis-dependent strand annealing (SDSA) but has only a limited role in single-strand annealing (SSA) [25]. Our previous study of OsRecQl4 showed that over-expression of OsRecQl4 promoted the resection process of HR-mediated DSB repair in rice [26]. However, the functions of BLM-related proteins in plants have not been well analyzed, particularly in the context of their potential "caretaker" role during DNA replication in plant meristems.

The presence of seven RecQ-like genes has been reported in rice—a monocotyledonous model plant. However, these seven genes in rice do not correspond to the seven Arabidopsis genes. Interestingly, a single BLM counterpart gene, *OsRecQl4*, has been predicted in the rice genome [8,27]. Although the presence of two BLM-like genes with different functions in Arabidopsis is interesting from the perspective of the evolution of genome stability in plants, the presence of a single *BLM* counterpart gene in rice seems to favor the functional analysis of BLM counterpart genes. Several previous reports in *Arabidopsis* had shown the effect of *BLM* ortholog genes on HR; we asked whether this function could have some relationship to the maintenance of genome stability during DNA replication.

In addition to having an advantage for the analysis of *BLM* counterpart genes in plants as mentioned above, rice is an ideal model plant in which to evaluate the functions of genes involved in genome stability at sites of DNA replication, i.e., the meristem. Furthermore, S-phase is concentrated in the region of rice root meristems [28]. This is because, in rice, the genotoxic stresses that accompany the arrest of DNA replication never induce endoreduplication [29], which is one of the routes of escape from DNA damage used in *Arabidopsis*[30].

Here, we report the cloning of *OsRecQl4* from rice and characterize the phenotypes of knockout plants in terms of DNA stability at the site of DNA replication. Our findings indicate that OsRecQl4 plays an important role in maintaining genome stability, at least in the root apical meristem (RAM), via suppression of HR.

## Results

### Isolation of *OsRecQl4* cDNA from rice

The presence of an AtRecQ4A like sequence in the rice genome was reported by Hartung and Puchta [8], who identified the 1164-amino acids protein CAE03209 (GenBank ID) as OsRecQ4A. We cloned the corresponding full-length cDNA by RT-PCR and RACE using specific primers designed according to the genomic sequence Os04g0433800 containing the CAE3209 coding sequence. Our cDNA sequence encodes 1174 amino acids and is the same as the Loc_Os04g35420.1 sequence predicted in the GRAMENE database [http://www.gramene.org/]. We named our sequence OsRecQl4 for *Oryza sativa* RecQ like protein 4.

**Figure 1:**
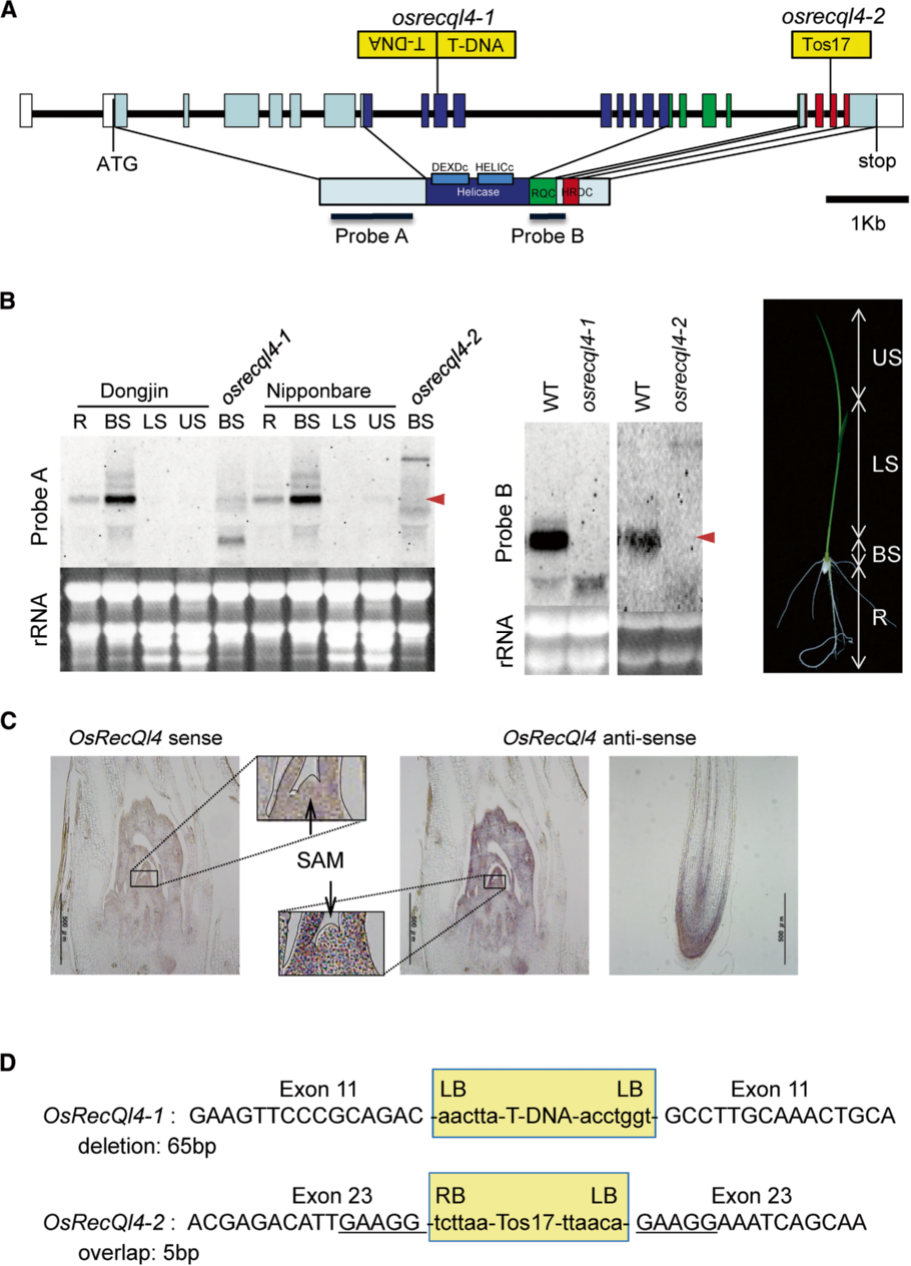
**Characterization of OsRecQl4 and molecular analysis of knockout lines.** (**A**) T-DNA (from POSTECH) insertion site in *osrecql4-1*, and Tos17 (from NIAS) insertion site in *osrecql4-2*. *OsRecQl4* has conserved helicase domains (including DEXDc, and HELICc domain), RecQ C-terminus (RQC), and HRDC domain. Probe A (near the N-terminus, 994 bp), and probe B (part of the RQC domain, 433 bp) were used for northern blot analysis. The T-DNA line shown above the construct harbored a head-to-head insertion in exon 11. The Tos17 line carries an insertion in exon 23. (**B**) Northern blot analysis. *OsRecQl4* mRNA is expressed strongly in the base of shoots (BS) and weakly in roots of both Dongjin and Nipponbare. mRNA was detected by both probes A and B in wild-type (WT) plants but not in *osrecql4-1* or *osrecql4-2.* Red arrowheads indicate mRNA of *OsRecQl4*. The genetic background of T-DNA (*osrecql4-1*) is Dongjin, and that of Tos17 (*osrecql4-2*) is Nipponbare. 7-day-old seedlings of wild-type (segregated wild type) and mutant (homozygous). R, root; BS, base of shoot; LS, lower shoot; US, upper shoot. **(C**) *In situ* hybridization analysis. Anti-sense probes for OsRecQl4 gave strong hybridization signals around the shoot apical meristem (SAM) and the root apical merstem (RAM). OsRecQl4 is expressed mainly in the meristem. (**D**) Sequence of the *osrecql4-1* and *osrecql4-2* insertion site. *osrecql4-1* has a genomic deletion of 65 bp in the insertion region, exon 11. *osrecql4-2* has a genomic overlap of 5 bp in the insertion region, exon 23.

*OsRecQl4* consists of 25 exons and 24 introns, and encodes a protein with 1174 amino acids including the DEXDc (DEAD-like helicase domain including ATP−Mg^++^ binding site) and HELICs (helicase superfamily c-terminal including ATP, nucleotide binding site) helicase domains, as well as an RQC (RecQ C-terminal) and an HRDC (required for dissolution of double Holliday junctions) domain [6] (Figure 1A). The result is the same as the prediction by the Rice Genome Annotation Project [http://rice.plantbiology.msu.edu/cgi-bin/ORF_infopage.cgi?orf=LOC_Os04g35420.1]. Because the DEXDc domain is conserved in BLM and AtRecQ4A, the OsRecQ4A (CAE3209) predicted by Hartung and Puchta lacking the DEXDc domain seems to be non-functional (Additional file 1: Figures S1 and S2).

*Arabidopsis* AtRecQ4A and AtRecQ4B are reported to be paralogous proteins with different functions. Since the amino acids sequences of AtRecQ4A and OsRecQl4 are quite similar to each other, and differ slightly from that of AtRecQ4B protein, we hypothesized that AtRecQ4B protein has acquired or lost specific motifs required for different functions. We compared several motifs of OsRecQl4, AtRecQ4A and AtRecQ4B proteins using the SALAD database [http://salad.dna.affrc.go.jp/salad/en/] and found that, near the C-terminus of the helicase domain, a CMKMGYNC sequence was detected only in AtRecQ4B; and in the N-terminus of the helicase domain, a DKESQKSQFLSSTATRI sequence is conserved between OsRecQl4 and AtRecQ4A but not in AtRecQ4B (data not shown). Further investigations of these motifs are required. It might be also interesting to try to express either AtRecQ4A or AtRecQ4B protein in the *osrecql4* mutant background.

Phylogenetic analysis revealed the isolated OsRecQl4 to be most similar to AtRecQ4A (51.5%) and AtRecQ4B (51.0%) at the amino acid level (Additional file 1: Figure S3A). The amino acid sequence encoded by the region spanning exon 8 to exon 16 of OsRecQl4 had especially high identity to that encoded by the region spanning exon 9 to exon 17 of AtRecQ4A (data not shown). As expected, OsRecQl4 more closely resembled AtRecQ4A than AtRecQ4B as determined by BLASTZ alignment analysis [31] (Additional file 1: Figure S3B).

### Expression of *OsRecQl4* in meristems

Expression of *OsRecQl4* was analyzed by northern blot in 7-day-old seedlings of the japonica-type rice cultivars Dongjin or Nipponbare. Using probe A (Figure 1A), relatively strong signals were observed in the basal parts of shoots (BS, as defined in Figure 1B) including the shoot apical meristem (SAM), and weak signals were observed in the root (R). No signal was detected in the lower (LS) or upper (US) shoot (Figure 1B).

In addition, SAM- and RAM-specific expression of *OsRecQl4* was confirmed by *in situ* hybridization (Figure 1C). Interestingly, meristem-specific expression of other RecQ family genes in rice was reported by Saotome et al. [27], who analyzed four other RecQ members—*OsRecQ1*, *OsRecQ2*, *OsRecQsim* and *OsRecQ886*—in rice. Our result was further supported by a database analysis of RiceXpro [http://ricexpro.dna.affrc.go.jp/ ]—a repository of gene expression profiles derived from microarray analysis of tissues or organs encompassing all growth stages of rice plants [32]. *OsRecQl4* was highly expressed in the RAM (Additional file 1: Figure S4). These results suggested that expression of *OsRecQl4* was restricted to meristematic tissues.

To elucidate the biological role of OsRecQl4, we characterized T-DNA and Tos17 retrotransposon inserted lines of OsRecQl4, namely *osrecql4-1* (3A-03503 from POSTECH) of Dongjin background and *osrecql4-2* (NC2763 from NIAS) of Nipponbare background, respectively. The sequences of the insertion sites were determined by PCR and subsequent sequencing. Two T-DNAs linked in a head-to-head manner were inserted into exon 11 with a genomic deletion of 65 bp. The Tos17 line was inserted at exon 23 accompanied by 5 bp overlap (Figure 1A and D).

Expression of OsRecQl4 in *osrecql4-1* and *osrecql4-2* mutants was evaluated by northern blot analysis. In this experiment, total RNAs from BS in 7-day-old WT and *osrecql4* mutants were analyzed. Probe A was located near the N-terminus and probe B was located near the C-terminus and included part of the RQC domain (Figure 1A). A transcript of ca. 3.5 kb was detected by probe A and B in WT plants, but not in *osrecql4-1* or *osrecql4-2* (Figure 1B). Under normal conditions, growth of *osrecql4-1* and *osrecql4-2* plants was comparable to that of WT plants. Furthermore, these mutant plants were fertile, as also reported for Arabidopsis *atrecq4A* mutants [23].

### OsRecQl4 is involved in DSB repair and recovery from S-phase arrest

**Figure 2:**
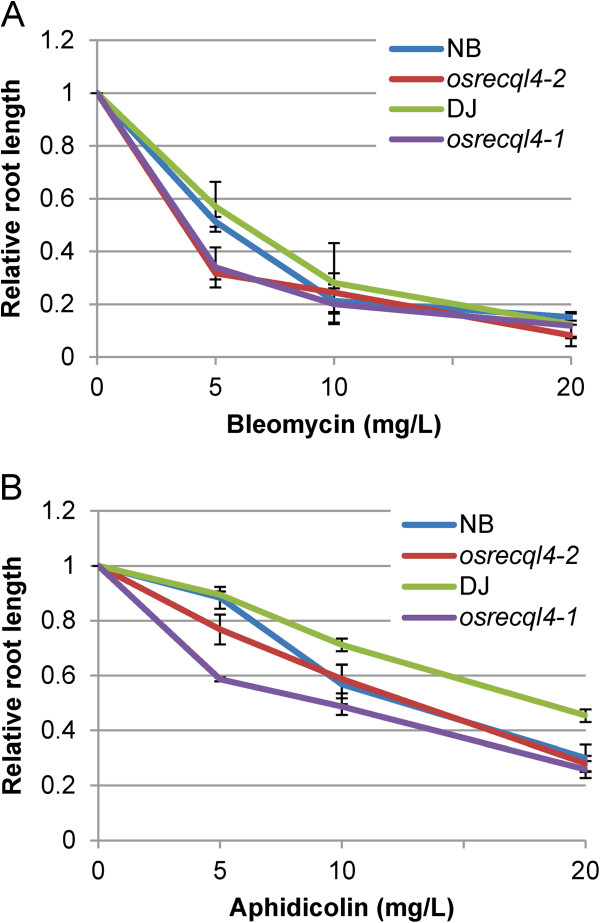
**Both *osrecql4-1 *and *osrecql4-2 *mutant plants show hypersensitivity to DNA damaging agents.** Both *osrecql4-1* and *osrecql4-2* mutant plants exhibit significantly reduced root length compared to WT plants upon treatment with (**A**) 5 mg/L bleomycin (DSB inducer) or (**B**) 5 mg/L aphidicolin (S-phase arrestor). After germination, 5-day-old seedling were grown on MS medium containing bleomycin or aphidicolin. WT: DJ (Dongjin), NB (Nipponbare) and mutants segregated out: the total length of roots of *osrecql4-1* (T-DNA), *osrecql4-2* (Tos17) were measured. The results shown are from three independent repeats. From 10 to 15 seedlings were tested per treatment.

To assess sensitivity to DNA damage, seeds from *osrecql4* mutants and their WT counterparts were treated with bleomycin at concentrations between 0 and 20 mg/L. Five days after germination, total root length was measured. When *osrecql4-1* or *osrecql4-2* mutants were treated with 5 mg/L bleomycin, root length was shorter than that of WT plants, suggesting that OsRecQl4 might be involved in the repair of DNA single-strand breaks (SSBs) and/or DSBs (Figure 2A). Similarly, we assessed the involvement of OsRecQl4 in the recovery process from S-phase arrest caused by inhibition of DNA polymerase. DNA replication can be arrested by aphidicolin—an inhibitor of DNA polymerase α [33]. Decreased root growth was observed upon treatment with 5 mg/L aphidicolin (Figure 2B). A similar result was obtained upon treatment with 2.5 mM hydroxyurea (data not shown). The expression of OsRecQl4 in roots increased in response to aphidicolin but not bleomycin (Additional file 1: Figure S5).

**Figure 3:**
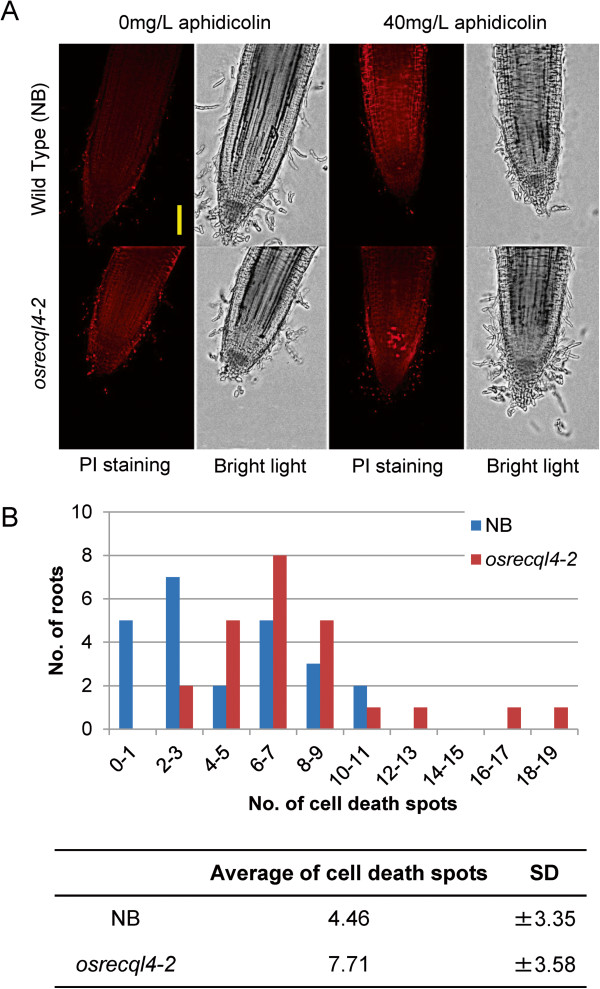
**Increased frequency of cell death induced in root tips of *osrecql4 *mutants upon aphidicolin treatment.** Propidium iodide (PI) staining indicates dead cells. (**A**) WT and *osrecql4-2* mutant plants treated with 40 mg/L aphidicolin for 24 h. An increased number of cell death signals was observed in the root meristem of *osrecql4-2* under the fluorescence microscope. Scale bar, 100 μm. (**B**) Summary of number of PI signals. 24 independent roots from *osrecql4-2* and WT plants were investigated. The x-axis indicates number of cell death spots per root. SD, standard deviation.

Judging from the result of northern blot analysis, both *osrecql4-1* (T-DNA line) and *osrecql4-2* (Tos17 line) mutants have non-functional protein if expressed since *OsRecQl4* transcripts were not detected with probe B. Furthermore, both mutants showed hyper-sensitivity against bleomycin and aphidicolin (Figure 2). In addition, we detected increased cell death upon aphidicolin treatment not only in *osrecql4-2* (Figure 3) but also in *osrecql4-1* (Additional file 1: Figure S7) in our previous manuscript.

Since *osrecql4-1* mutant has a GUS expression cassette in its T-DNA region, we could not evaluate homologous recombination frequency using the GU-US recombination substrate system. Therefore, we used mainly the *osrecql4-2* mutant line for our experiments.

### Failure to recover from DNA replication arrest induces DSBs

In humans, BLM is involved in the recovery process from DNA replication arrest that can be induced by SSBs or DNA adducts by the unwinding of arrested replication forks. In addition, the increased SCE observed in *blm* mutant cells is explained by enhanced DSBs due to a lack of the helicase activity of BLM [4]. We therefore investigated whether the increased sensitivity of *osrecql4* mutants to aphidicolin treatment was due to a defect of the recovery from DNA replication arrest in *osrecql4* cells as like *blm* mutant cells and a detected increased DSBs in *osrecql4* plants.

**Figure 4:**
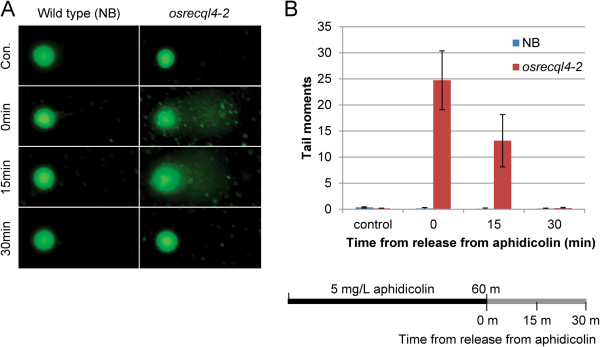
**DSBs are induced in *osrecql4-2 *mutants upon aphidicolin treatment.** The comet assay, which can detect DNA breaks at the single cell level, detected DSBs under a neutral conditions protocol. Without aphidicolin treatment, no broken DNA fragments were observed in either wild-type or *osrecql4* cells. Upon treatment with 5 mg/L aphidicolin for 60 min, broken DNA fragments were observed in *osrecql4-2* cells under the fluorescent microscope (**A**) as summarized in (**B**) for *osrecql4-2*. The DSBs almost disappeared in *osrecql4-2* after transferring to aphidicolin-free MS medium for 30 min. Immediately after treatment of aphidicolin; *15* aphidicolin-free MS medium for 15 min after treatment with aphidicolin; *30* aphidicolin-free MS medium for 30 min after treatment of aphidicolin.

We used a comet assay to investigate induction of DSBs following aphidicolin treatment. Six-week-old scutellum-derived calli from Nipponbare (NB) and *osrecql4-2* were treated with 5 mg/L aphidicolin for 60 min; the cells were then observed over time after removal of the aphidicolin. The comet tail of *osrecql4-2* did not differ from that of control NB without aphidicolin treatment. Following aphidicolin treatment, comet tails indicating DSBs were seen in *osrecql4-2* cells immediately after aphidicolin removal, and remained at 15 min after removal of aphidicolin. In contrast, no comet tails were detected in control NB cells, even after aphidicolin treatment (Figure 4). These results suggest that, in the absence of OsRecQl4, aphidicolin-triggered DNA replication arrest resulted in DSB induction. Although these DSBs might be finally repaired by intrinsic DNA repair systems including HR, cell cycle progression will be delayed by the time needed for DSB repair.

**Figure 5:**
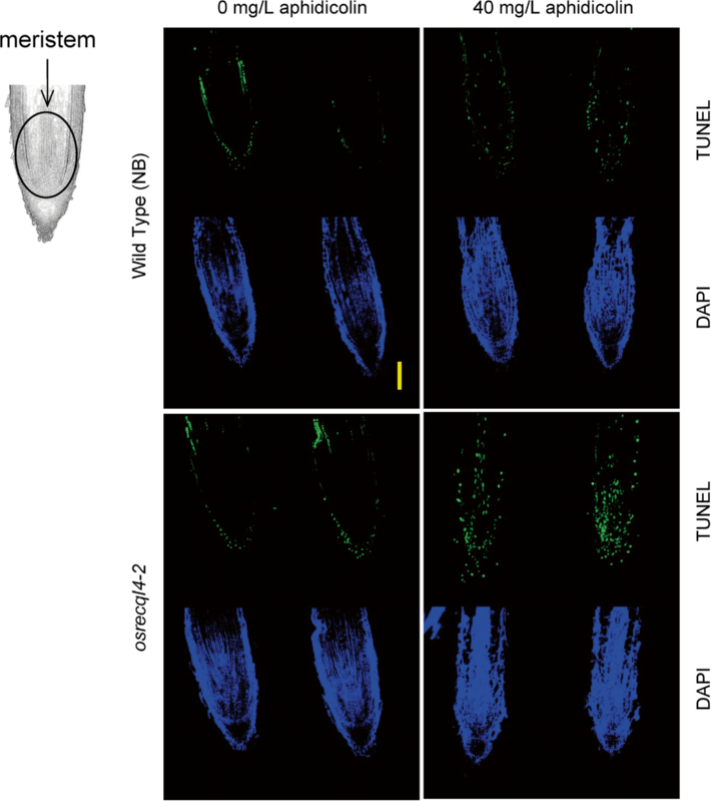
**DNA breaks are induced in the root meristem of *osrecql4-2 *mutant plants upon aphidicolin treatment.** The TUNEL assay detects single- and double-strand DNA breaks and nicks via 3'-OH end labeling. Following 40 mg/L aphidicolin treatment for 24 h, the number of DNA damage signals seen in root meristems of *osrecql4-2* under the fluorescent microscope was observed. DNA damage signals in the epidermal cells seem to be induced by light or physical stress. Scale bar, 100 μm.

Next, we used TUNEL (terminal deoxynucleotidyl transferase dUTP nick-end labeling) to observe the occurrence of DNA damage in root tips of the *osrecql4-2* mutant after aphidicolin treatment. This assay detects DSBs, SSBs, and nicks in tissues by labeling the 3-terminal ends of nucleic acids [34,35]. A DNA damage signal was observed in the meristem and in epidermal cells (Figure 5). The former signal is related to cell division but the latter seems to be induced by physical stress during root growth. TUNEL signals induced by physical stress have been observed in root caps and root hairs [36]. DNA damage signals were observed in the root meristem of *osrecql4-2* plants but not in WT plants after treatment with 40 mg/L aphidicolin (Figure 5). These results indicate that enhanced DNA replication arrest induced DNA damage in the RAM of the *osrecql4-2* mutant.

### Accumulated DSBs triggered by DNA replication arrest induce HR

Induced DSBs can be repaired by intrinsic DNA repair systems; if they are not repaired properly, cell death might be induced. Because HR is a DSB repair system used during S-phase of the cell cycle, when sister chromatids are available as templates for DNA repair, we analyzed whether the induced DSBs observed in *osrecql4* mutants could enhance HR.

**Figure 6:**
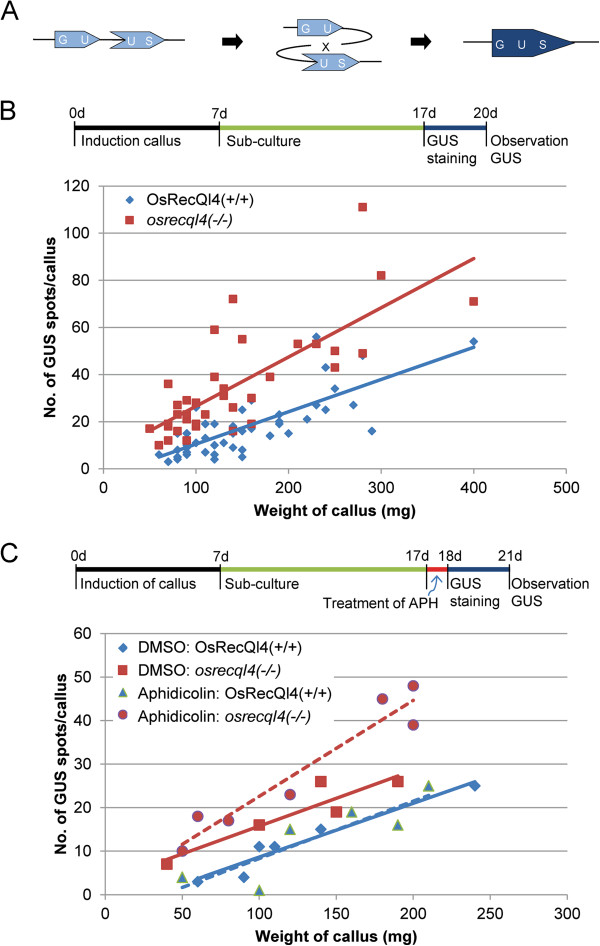
**Enhanced HR in *osrecql4-2 *mutants.** (**A**) The GUS recombination reporter (pGU.C.US) used in this experiment is illustrated schematically. Recombination between the two overlapping sequences produces a functional GUS gene. (**B**) Graph showing the number of GUS blue spots per callus weight as counted under the microscope. HR frequency increased more in the *osrecql4-2* mutant than in the WT. GUS histochemical staining was performed on 17-day-old calli. **(C**)Aphidicolin treatment enhanced HR further in *osrecql4-2* mutants, but HR frequency in WT did not differ from that of the WT without aphidicolin treatment. 17-day-old calli were treated 5 mg/L aphidicolin for 24 h. The aphidicolin-treated calli were then subjected to GUS staining. *Blue diamonds* OsRecQl4(+/+); *red squares* OsRecQl4(−/−); *blue solid line* average of OsRecQl4(+/+) GUS spots; *red solid line* average of OsRecQl4(−/−) GUS spots; *blue triangles* aphidicolin treated OsRecQl4(+/+), *red circles* aphidicolin treated OsRecQl4(−/−); *blue dotted lines* average of aphidicolin-treated OsRecQl4(+/+) GUS spots; *red dotted line* average of aphidicolin-treated OsRecQl4(−/−) GUS spots.

We used the GUS recombination reporter pGU.C.US [37] to monitor the *osrecql4* mutant for frequency of HR (Figure 6A). Recombination events between two overlapping GUS sequences can produce a functional GUS gene resulting in blue spots in the cells that can be detected by GUS histochemical staining with 5-bromo-4-chloro-3-indolyl glucuronide. pGU.C.US was transformed into a heterozygous *osrecql4-2* line; a transgenic line with a single copy of the recombination reporter was identified by Southern blot analysis (Additional file 1: Figure S6) and used for further studies. The frequency of HR events was monitored in the T1 generation of pGU.C.US transgenic rice plants, which were homozygous for either the *osrecql4* mutation allele or the WT *OsRecQl4* allele. We evaluated the frequency of HR events in scutellum-derived calli.

The distribution of HR frequency by callus weight indicated that HR frequency was greater in the *osrecql4-2* mutant than in WT (Figure 6B). HR frequency did not change in WT but increased further in the *osrecql4-2* mutant upon aphidicolin treatment (Figure 6C).

### Un-repaired DNA replication arrest induces cell death

The increased sensitivity to aphidicolin observed in *osrecql4* mutants implied that the increased number of DSBs detected in these mutants could induce delayed cell cycle progression or that DSBs might not have been repaired sufficiently by HR.

We revealed cell death by the uptake of PI, which permeates the cell membranes of dying and dead cells [38]. PI-stained root tips were detected after aphidicolin (40 mg/L) treatment for 24 h. We observed root tips using the Z-stack function (see Microscopic analysis in the Methods section). Upon aphidicolin treatment, we detected a significantly increased number of PI staining signals in *osrecql4-2* mutants (7.71 ± 3.58) than in WT NB (4.46 ± 3.35 ; P<0.01, Mann–Whitney *U* test) (Figure 3). Similarly, we observed increased PI staining signals in *osrecql4-1* mutants (1.47 ± 0.18) than WT Dongjin (0.20 ± 0.14) (Additional file 1: Figure S7). The optimum concentration of aphidicolin for the visualization of dead PI-stained cells differed with ecotype. We also used PI staining to see if other types of inducers of DNA damage affected induction of cell death; however, no other inducers led to significant differences between WT and *osrecql4* (Additional file 1: Figure S8A). Nevertheless, high concentrations or prolonged treatment induced abnormal shapes in the roots and turned them opaque (Additional file 1: Figure S8B). This result suggested that *osrecql4* mutant plants failed to recover from DNA replication arrest, resulting in an increased numbers of DSBs, leading to induction of cell death in the root meristem.

## Discussion

Here, we assayed the function of the BLM homolog OsRecQl4 during DNA replication in rice via knockout mutants. Using the comet assay, we demonstrated induction of DSBs in aphidicolin-treated *osrecql4* mutant cells. TUNEL analysis suggested that DNA damage including DSBs was induced at the RAM. The HR assay using the GUS recombination reporter showed that at least a fraction of the DSBs could be repaired by HR, explaining why HR is enhanced in *osrecql4* mutants. The PI staining assay showed that un-repaired DSBs induced cell death at the root meristem. The combined results of comet, TUNEL and HR assays as well as PI staining using mutant plants under a combination of aphidicolin treatments clearly demonstrated the important role of OsRecQl4 in the process of recovery from DNA replication arrest, and also the conserved role of BLM orthologous proteins in this process. In this study, we focused on the role of OsRecQl4 in genomic maintenance during DNA replication in the RAM. However, OsRecQl4 is also expressed in the SAM (Figure 1B, C). Thus, we consider OsRecQl4 to be involved in the maintenance of genome stability during DNA replication at the SAM as well as the RAM. Therefore, increased mutations could accumulate in *osrecql4* mutant plants during the mitotic cell cycle, and these mutations should be inherited by subsequent generations.

The *osrecql4-1* (T-DNA line) has only a small amount of truncated protein and *osrecql4-2* (Tos17 line) has a longer protein according to northern blot analysis (Figure 1B). Thus, we concluded that both the *osrecql4-1* and the *osrecql4-2* mutants produce an aberrant size of OsRecQl4 protein defective in the consensus domain RQC if expressed. The two mutants might have different effects. However, we revealed both mutants have hyper-sensitivity to DNA damage agents and increase cell death upon aphidicolin treatment.

In this study, we have shown that DNA replication arrest leads to a hyper-recombination phenotype in plants. Urawa et al. [39] reported that the non-transcribed spacer (NTS) between ribosomal RNA genes (rDNA), which contains a replication fork barrier of rDNA [40], enhances HR in *Arabidopsis*. In *Escherichia coli*, it has been hypothesized that a damaged DNA replication fork might be restarted by Holliday junction formation, leading to DNA cleavage by Holliday junction dissolution and finally to repair by HR. However, this mechanism runs the risk of inappropriate recombination. Thus, a stalled fork (not leading to DNA cleavage) might be processed by DNA helicases to avoid replication errors [41].

Recently, Schuermann et al. [42] reported that a defect in DNA polymerase delta 1 (*POLδ1*) in *Arabidopsis* exhibited elevated HR frequency at stalled and collapsed replication forks. This report also supported our conclusion that DNA replication stress induces HR in plants. However, the molecular mechanism connecting DNA replication stress and enhanced HR remains obscure in plants. Here, we see a relationship between accumulated DSBs, HR and cell death accompanying DNA replication arrest at the site of the meristem.

Rad51-dependent repair HR includes break-induced replication (BIR), double-Holliday junction (dHJ), and SDSA. Rad51-independent SSA also repairs DSB [43]. It was been reported recently that, with a direct repeat GU-US recombination reporter, a functional GUS gene can be generated mainly by the SSA pathway, with SDSA playing only a minor role following DSB [44]. However, during replication, induced DSBs do not induce two free ends, but rather a one-ended DSB, i.e., a DNA double-strand end (DSE). The DSE invades its sister chromatid to be repaired, with DNA synthesis by BIR [45,46]. Thus, although increased HR efficiency was demonstrated here using the direct repeat GU-US recombination reporter to associate OsRecQl4 and HR, the HR detected should be Rad51-dependent HR repair. In future, this could be confirmed using a double mutant with Rad51. Further points in support of the hypothesis of alternative repair of the GU-US reporter are the differences in tissues (plant vs. calli) and induction (I-*Sce*I vs. aphidicolin), which might lead to different prevalence of cell-cycle states in which the repair takes place.

We analyzed the sensitivity of *osrecql4* mutants to aphidicolin and bleomycin. Since defects in OsRecQl4 enhance the sensitivity of rice to both these latter compounds, *OsRecQl4* might be involved in recovery from DNA replication arrest and DSB repair. On the other hand, *osrecql4* mutants showed normal growth and were fertile, although *osrecql4* mutants showed aberrantly sized OsRecQl4 protein, indicating that OsRecQl4 might not be essential. In this respect, it has been reported that members of RecQ family genes in rice—*OsRecQ1*, *OsRecQ2*, *OsRecQsim* and *OsRecQ886*—are expressed in meristems [27], suggesting that RecQ helicase family members might play overlapping roles in maintaining genome stability in proliferative cells. However, homozygous *blm* mice exhibit growth retardation [47]. It might be interesting to evaluate growth of the *osrecql4* mutant under conditions of elevated UV, since DNA replication arrest is induced by UV photoproducts.

Although the sensitivity of Arabidopsis *atrecq4A* mutants to bleomycin treatment was the same as that of WT plants, in our study we observed enhanced bleomycin sensitivity in rice *osrecql4* mutants compared to WT plants. This might be due to differences in the system, tissue and experimental procedure. Furthermore, we observed cell death in the root meristem of *osrecql4* mutant plants. These differences might be attributed to the occurrence of endoreduplication in *Arabidopsis*, since cells undergoing DNA damage can enter into endocycle and be separated from the mitotic cell cycle in *Arabidopsis*.

Our results indicate that OsRecQl4 is expressed at SAM and RAM, i.e., sites of cell division. Furthermore, expression of OsRecQl4 was induced by aphidicolin treatment but not by bleomycin (Additional file 1: Figure S5). These results suggest that, according to their level of transcription, OsRecQl4 plays a more important role in repair during DNA replication than in DSBs repair. BLM assembles at stalled replication forks [4,6], which supports the notion of DNA replication arrest inducible expression of OsRecQl4 (Additional file 1: Figure S5). These results suggest that OsRecQl4 is required for recovery from DNA replication arrest. The *osrecql4-2* mutant failed to recover from DNA replication arrest; this resulted in an increased number of DSBs requiring repair, possibly by HR.

Interestingly, we found that, without aphidicolin treatment, both WT and *osrecql4-2* mutants produced a very low level of DSBs (Figure 4) judging from the comet assay. Similarly, DNA damage in *osrecql4-2* at the cell division root zone analyzed by TUNEL was almost comparable to that of WT plants. However, the *osrecql4-2* mutant showed a greater frequency of HR than did WT plants under normal growth conditions without aphidicolin treatment. This suggested that DSBs induced in the *osrecql4-2* mutant could be repaired by DNA repair systems, including HR, under normal growth conditions, since HR can proceed slowly without RecQ helicase [48]. However, excess DSBs induced by aphidicolin treatment could not be repaired by intrinsic DNA repair systems, and un-repaired DSBs were thus detected by comet and TUNEL assay. Furthermore, un-repaired DSBs might induce cell death in *osrecql4* mutants upon aphidicolin treatment. Thus, OsRecQl4 might be required to rescue plants from genotoxic stress.

Broadly speaking, RecQ helicases maintain DNA stability. The RecQ helicase BLM promotes not only recovery from DNA replication arrest, but also exonuclease 1-mediated DNA resection during the initial step of DSB repair [49]. OsRecQl4 also plays a role in promoting processing of HR-mediated DSB repair in rice [26]. OsRecQl4 was expressed exclusively in meristems; also, other rice RecQ-like genes were expressed in meristematic tissues [27]. Thus, we anticipate overlapping and distinct roles for the seven RecQ helicase genes in rice.

## Conclusions

The results reported here indicate that OsRecQl4 has an important function in maintaining genome stability in rice during DNA replication. Since BLM and SGS1 exert their function of recovering arrested replication forks by their helicase activity, OsRecQl4 might have the same activity in genome maintenance in rice. In particular, enhanced DSBs and cell death were observed upon DNA replication arrest in the cell division zone of *osrecql4* mutants. From this, we conclude that OsRecQl4 is required for recovery from DNA replication arrest in the rice meristem. RecQ helicases should have partially overlapping roles in maintaining genome stability, and among them might be specialists whose primary role is genome stability.

## Methods

### Phylogenetic analysis

We searched the rice full-length cDNA databases, NCBI [http://www.ncbi.nlm.nih.gov/] and GRAMENE [http://www.gramene.org/], using the programs BioEdit (version 7.0.5.3) and MEGA (version 3.1) to identify rice homologues of RecQ. Seven RecQ homologues are found in rice. Phylogenetic analysis was performed based on the amino acid sequence alignment generated by the built-in CLUSTALW program.

### Synthesis of OsRecQl4 cDNA and isolation of *osrecql4* mutant lines

Total RNA was isolated from 7-day-old seedlings using an RNeasy Plant Mini kit (Qiagen) according to the protocol supplied by the manufacturer. Reverse transcription was performed using ReverTra Ace (Toyobo, Japan) according to the instructions provided by the manufacturer, with total RNA from rice shoot base. Polymerase chain reaction (PCR) and rapid amplification of cDNA ends (RACE) were performed using high fidelity thermostable DNA polymerase, KOD-Plus (Toyobo, Japan). 5’- and 3’-RACE were performed using a GeneRacer kit (Invitrogen, Carlsbad, CA) with gene-specific primers designed according to the genomic DNA sequence. The gene specific primers used for amplification of the coding region of OsRecQl4 were RecQl4_Start1 (GCCATGATAAAGCCAAGGGTCAACT), and primers for amplification of full length cDNA were RecQl4_End1 (ACCCTAGGCTATTCTGGCGGACTG), RecQl4-5’Race (GATCCGACCAGTTGACCCTTGGCTTT) and RecQl4-3’Race (ACGCGCTGCAAAGACACGTACAAGG). PCR products were cloned into pCR2.1 TOPO vector (Invitrogen, San Diego, CA) for DNA sequencing.

Seeds of the T-DNA tagged line *osrecql4-1* and of the Tos17 insertion line *osrecql4-2* were obtained from POSTECH (line No. 3A-03503) [50] and NIAS collections (line No. NC2763), respectively [51]. Seeds derived from heterozygous plants were cultivated in soil, and those for the T-DNA and Tos17 insertion lines were propagated further in the greenhouse.

The sites of integration of T-DNA and Tos17 were determined by PCR using the following primers: for T-DNA insertion, combinations specific for the left or right border of the respective T-DNA: pGA2715-L1.5 (GGCCAGTGAATTCACTAGTGATTGC), 3A-03503-498 F (CAATCCATCCTCGAAAGGCAAT), 3A-03503-498R (ATTCGCGAGGCCATCTCTCT) and for Tos17 insertion: Tos17_3880 (AGTCGCTGATTTCTTCACCAAGG), NC2763-F (TGCCTTGTACGTGTCTTTGC), NC2763-R (AGCTTTGCAATGCCTTAGGA) and genomic sequences within the respective gene. PCR products were purified and sequenced (Figure 1A).

### Northern blot analysis

Total RNA was prepared from the shoot tips of 10-day-old seedlings using an RNeasy Plant Mini Kit (Qiagen, Valencia, CA). Total RNA (20 μg) was loaded and separated on a 1% agarose gel, and transferred onto a positively charged nylon membrane (Roche, Mannheim, Germany). Probe A (OsRecQ4-proN_F, AACACAAAGGCCTAATCAGGAAGCA; OsRecQ4-proN_R, ATTCCTGGTGTAAATCGGTGATTGG) or B (RecQl4_1F, GTCGAAAAAGATGTGACCAACATTGCTAG; RecQl4_1R, TCCCGTCCACTTGACTCTGTTGATTAG) (Figure 1A) was prepared using a PCR DIG probe synthesis kit (Roche), and hybridization was performed according to the DIG Application Manual (Roche). Hybridization was performed at 50°C, and washing was performed under high-stringency conditions at 50°C.

### Treatment with DNA damaging agents

To study the effect of DNA damaging agents on rice, We prepared a 10 mg/ml stock of cisplatin (CDDP, cis-diamminedichloroplatinum [II]; Wako Pure Chemical Industries, Tokyo, Japan), a 10 mg/ml stock of bleomycin (BLE; Wako Pure Chemical Industries), a 10 mg/ml stock of aphidicolin (APH; Wako Pure Chemical Industries), a 10 mg/ml stock of nocodazole (NOC; Wako Pure Chemical Industries), and a 1 mM stock of camptothecin (CPT; Wako Pure Chemical Industries).

To analyze the effect of DNA damaging agents on root elongation, sterilized seeds were sown on MS solid medium containing DNA-damaging agents and grown in a growth chamber at 30°C under continuous light. Five days after sowing, root length was measured using Image J software (version 1.43).

To prepare plant materials for PI staining and TUNEL assay, we transferred 5-day-old rice plants grown on MS solid medium to MS liquid medium containing different concentrations of DNA-damaging agents and then incubated for a further 24 hours in the growth chamber. For comet assay, 4-week-old calli grown on N6D solid medium were transferred to N6D liquid medium containing DNA damaging agents and then incubated for a further 1 hour at RT.

### PI staining of root tip

Root tips, which were cut approximately 10 mm from the root tip, were stained with 5 mg/L PI (dissolved in sterile water) for 20 min on a slide glass.

### TUNEL (terminal deoxynucleotidyl transferase-mediated dUTP nick and labeling) analysis

Roots were fixed overnight with 4% (v/v) paraformaldehyde in PBS (pH 7.4) at 4°C. Next, fixed roots were dehydrated in a series of tert-butanol and embedded in paraplast for sectioning. The samples were rehydrated in a graded ethanol series to water to remove paraformaldehyde, before being treated with proteinase K (20 μg/ml proteinase K in 10 mM Tris–HCl, pH 7.5) at 37°C for 30 min, and then washed three times with PBS. TUNEL reaction was performed on a slide glass using the In Situ cell death detection kit with fluorescein (Roche) according to the manufacturer's instructions.

### Comet assay

Microscopic slides were precoated with a layer of 1% normal melting point agarose and thoroughly dried. Calli were chopped in 200 μl PBS containing 50 mM EDTA with razor blade. 30 μl of the resulting suspension of nuclei was mixed with an equal volume of liquid 1% low-melting-point agarose at 42°C and spread on microscope slides pre-coated with 1% normal-melting-point agarose. After solidification of the agarose containing nuclei, the nuclei were subjected to lysis in a high salt solution (2.5 M NaCl, 10 mM Tris–HCl pH 7.5, 100 mM EDTA) for 20 min at room temperature. Equilibration for 3 × 5 min in 1×TBE on ice was followed by electrophoresis at room temperature in 1× TBE buffer for 6 min. To clear gels of the starch grains that are present in the nuclear suspension, the slides were kept for 10 min in 1% Triton prior to dehydration for 2× 5 min in 70% ethanol and 96% ethanol and air drying. Dry agarose gels were stained with a 1:10,000 diluted SYBR green. After staining, the slides were captured under a fluorescent microscope using Leica DM5000 microscope software and DNA damage was quantitated using CometScore ™ software.

### Microscopy

Images of PI-stained root tips were captured by a Keyence (BZ-9000) microscope with Z-stack function. The Z-stack function moves the focal point of an objective lens through the Z-axis, captures several images, extracts only focused points from the images, and synthesizes the points into an omni-focal image (http://www.keyence.com/products/microscope/fluorescence/bz8000/bz8000_features_3.php). A Leica DM5000 microscope was also used for image-capture.

### Rice transformation

*Agrobacterium*-mediated transformation of rice (*O. sativa* L. cv. Nipponbare) was performed as described previously [52,53]. Hulled seeds were sterilized and germinated on N6D medium for 1 week. After co-cultivation of *Agrobacterium* carrying the pGU.C.US vector, infected calli were washed with sterilized water seven times and with 12.5 mg/L meropenem three times. The calli were transferred to N6D medium containing suitable antibiotics for selecting transformed cells. Calli that grew vigorously on the selection medium were transferred to regeneration medium containing antibiotics. The regenerated plants were further grown on hormone-free MS medium, and then planted in soil.

### Histochemical GUS staining

Histochemical staining of plant materials was performed as described by Jefferson [54]. To facilitate the penetration of staining buffer, plants were cut into segments of approximately 10 mm, and large calli were divided into several pieces. These plant materials were vacuum infiltrated with X-Gluc staining substrate for 3 × 10 min and then incubated at 37°C for 3 days. Calli were subsequently bleached with 70% ethanol and the number of GUS blue spots was counted.

## Abbreviations

BIR: Break-induced replication; BS: Base of shoot; dHJ: Double-Holliday junction; DSB: DNA double-strand break; DSE: Double-strand end; HR: Homologous recombination; LS: Lower shoot; NB: Nipponbare; NTS: Non-transcribed spacer; PI: Propidium iodide; POLδ1: DNA polymerase delta 1; R: Root; RAM: Root apical meristem; rDNA: Ribosomal RNA gene; RPA: Replication protein A; SAM: Shoot apical meristem; SCE: Sister chromatid exchange; SDSA: Synthesis-dependent strand annealing; SSA: Single-strand annealing; SSB: Single-strand break; US: Upper shoot.

## Competing interests

The authors declare that they have no competing interests.

## Authors’ contributions

YIK led the design of the study and carried out part of the experimental work, KA, ME participated in the design of the study, carried out part of the experimental work, and advised on experimental processes; KO participated in the study design, provided constructs of promoters and terminators etc.; AT carried out part of the *in situ* hybridization analysis; NO, ANY and HS participated in the study design, and carried out part of the experimental work. ST was overall study supervisor, participating in study design, helping write the manuscript and obtained the funding. All authors read and approved the final version.

## Supplementary Material

Additional file 1 OsRecQl4-replication-SD-r. Click here for file
