# Supplementary material for: DNA replication arrest leads to enhanced homologous recombination and cell death in meristems of rice OsRecQl4 mutants
Source: BMC Plant Biol. 2013 Apr 12;13:62. doi: 10.1186/1471-2229-13-62 (PMC3648487; doi:10.1186/1471-2229-13-62)
Supplement: Additional file 1 — OsRecQl4-replication-SD-r. [file 1471-2229-13-62-S1.pdf]

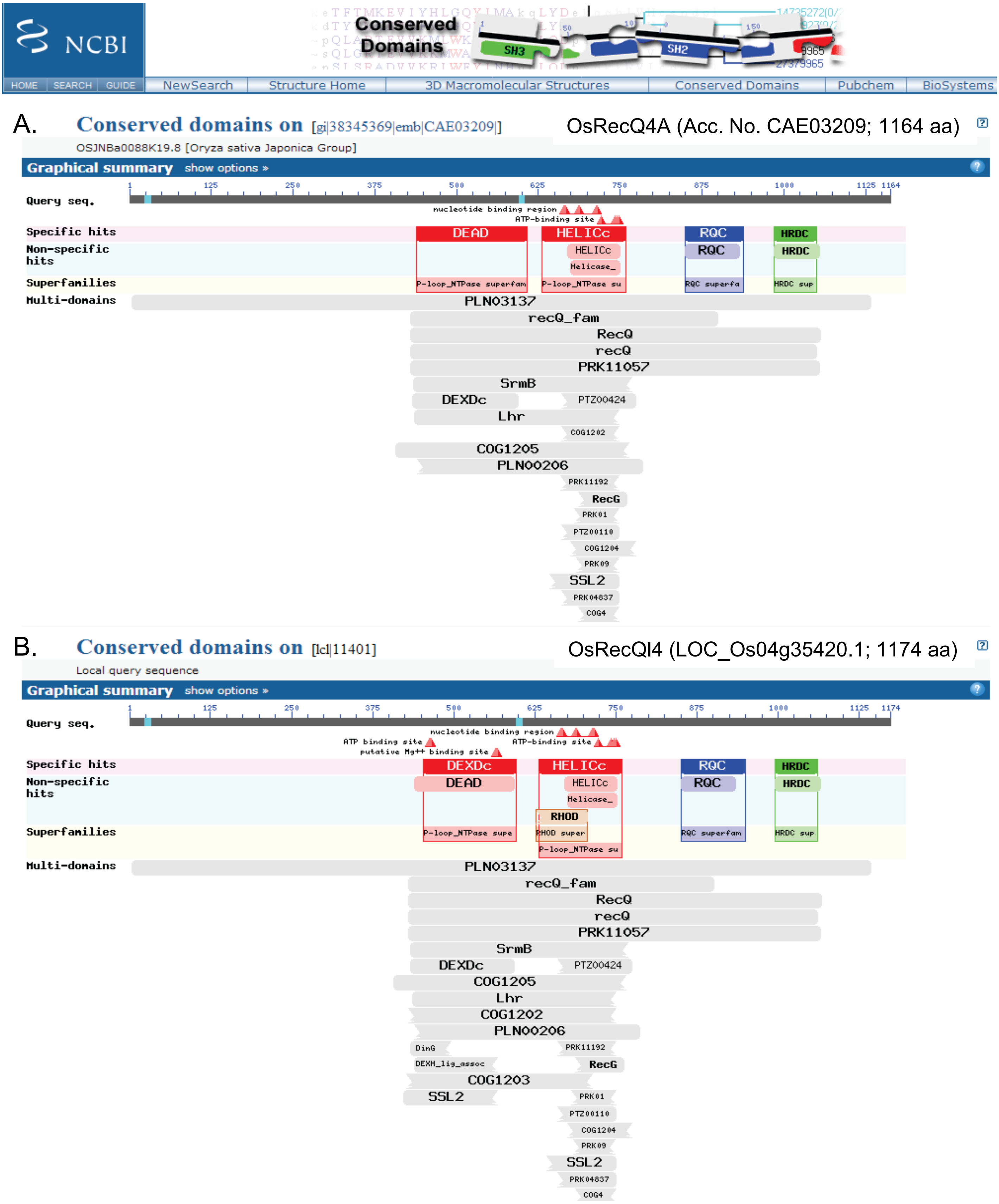

Supplemental Fig. 1. Conserved domians on OsRecQ4A and OsRecQl4 by NCBI database

(A) OsRecQ4A (Acc. No. CAE03209; 1164 aa) has not a DEXDc domain.

(B) OsRecQl4 (LOC_Os04g35420.1; 1174 aa) has a DEXDc domain. Which is DEAD-like helicases superfamily. A diverse family of proteins involved in ATP-dependent RNA or DNA unwinding. Thi s domain contains the ATP-binding region.

```
               ....|....| ....|....| ....|....| ....|....| ....|....|
                       410        420        430        440        450
OsRecQl4       IDISYTEGSG DKQWSSTHFA WTKELEANNK RVFGNRSFRP NQREIINATM
OsRecQ4A       IDISYTEGSG DKQWSSTHFA WTKELEANNK RVFGNRSFRP NQREIINATM

               ....|....| ....|....| ....|....| ....|....| ....|....|
                       460        470        480        490        500
OsRecQl4       SGNDVFVLMP TGGGKSLTYQ LPALICNGVT LVVSPLVSLI QDQIMHLLQA
OsRecQ4A       SGNDVFVLMP TGGGKSLTYQ S----QNVSI FEVG--TGIK RVGRNDWRKA

               ....|....| ....|....| ....|....| ....|....| ....|....|
                       510        520        530        540        550
OsRecQl4       NISAAYLSAS MEWSEQQEIL RELMSPTCTY KLLYVTPEKI AKSDALLRQL
OsRecQ4A       NISAAYLSAS MEWSEQQEIL RELMSPTCTY KLLYVTPEKI AKSDALLRQL

               ....|....| ....|....| ....|....| ....|....| ....|....|
                       560        570        580        590        600
OsRecQl4       ENLYSRGHLS RIVIDEAHCV SQWGHDFRPD Y-----QHLG ILKQKFPQTP
OsRecQ4A       ENLYSRGHLS RIVIDEAHCV SQWGHDFRPD YQFFIHQHLG ILKQKFPQTP

               ....|....| ....|....| ....|....| ....|....| ....|....|
                       610        620        630        640        650
OsRecQl4       VLALTATATA SVKEDVVQVL GLANCIIFRQ SFNRPNLRYF VWPKTKKCLE
OsRecQ4A       VLALTATATA SVKEDVVQVL GLANCIIFRQ SFNRPNLRYF VWPKTKKCLE
```

Supplemental Fig. 2. Sequence of DEXDc domain on OsRecQl4 DEAD-like helicases superfamily. Red box, DEXDc domain sequence (from 453 to 603). Gray box, different sequence between OsRecQl4 (1174 aa) and OsRecQ4A (1164 aa).

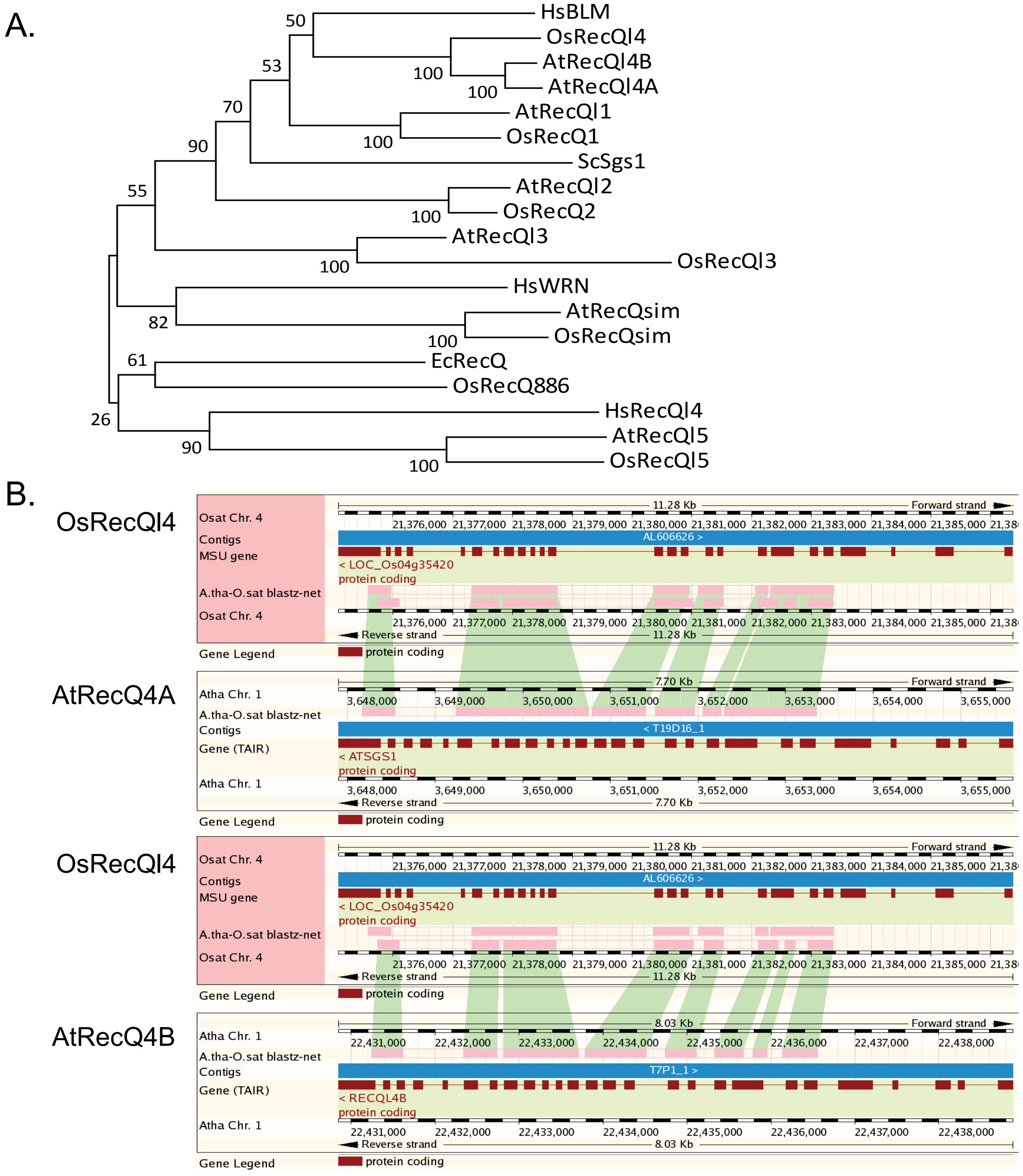

Supplemental Fig. 3. Phylogenetic analysis of RecQ-like proteins

(A) Phylogenetic analysis indicated that OsRecQl4 protein is most closely related to AtRecQl4A (51.5% identity), AtRecQl4B (50.0% identity), and HsBLM (39.0% identity). AtRecQl1 and OsRecQl1 do not contain the HRDC domain, which function in the dissolution of double Holliday junctions. EcRecQ is categorized in a separate group, because it is multifunctional. HsRecQl4 is not a counterpart of OsRecQl4. The open boxes represent the RecQ family in rice. A phylogenetic tree was constructed by the CLUSTALW method, based on the amino acid sequences of OsRecQ homologues and other known RecQ helicases. Branch lengths are proportional to evolutionary divergence expressed as substitutions per site. At, *Arabidopsis thaliana*; Os, *Oryza sativa*; Hs, *Homo sapiens*; Sc, *Saccharomyces cerevisiae*; Ec, *Escherichia coli*

(B) BLASTZ alignment analysis performed using GRAMENE (http://www.gramene.org). The green regions show conserved sequence between OsRecQl4 and AtRecQ4A/AtRecQ4B. As shown, OsRecQl4 shares more similarity with AtRecQ4A than with AtRecQ4B

http://ricexpro.dna.affrc.go.jp/RXP_4001/graph-view.php?featurenum=10034

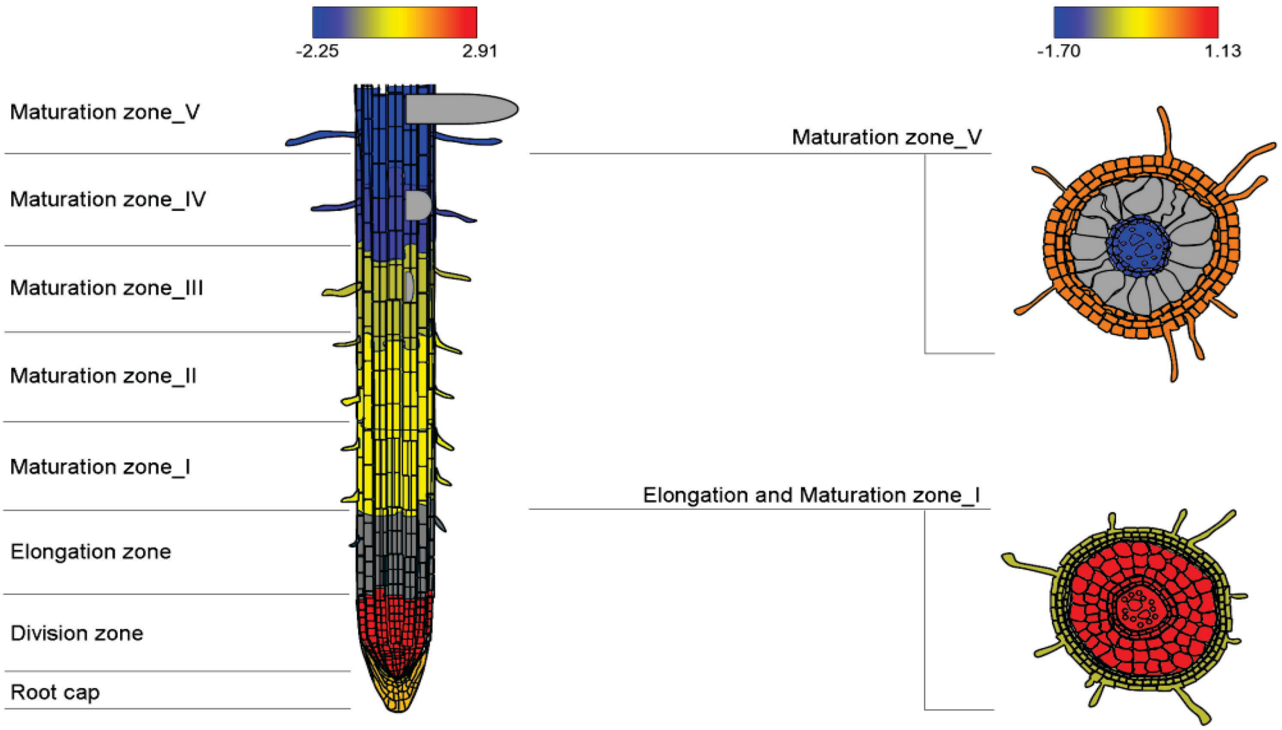

Supplemental Fig. 4. Expression of OsRecQl4 in root tip

The RiceXpro database (http://ricexpro.dna.affrc.go.jp/) is a repository of gene expression profiles derived from microarray analysis of tissues/organs encompassing the entire growth cycle of the ric e plant. Analysis revealed that OsRecQl4 is highly expressed in the RAM. Blue area, low expressio n of OsRecQl4; Red area, high expression of OsRecQl4.

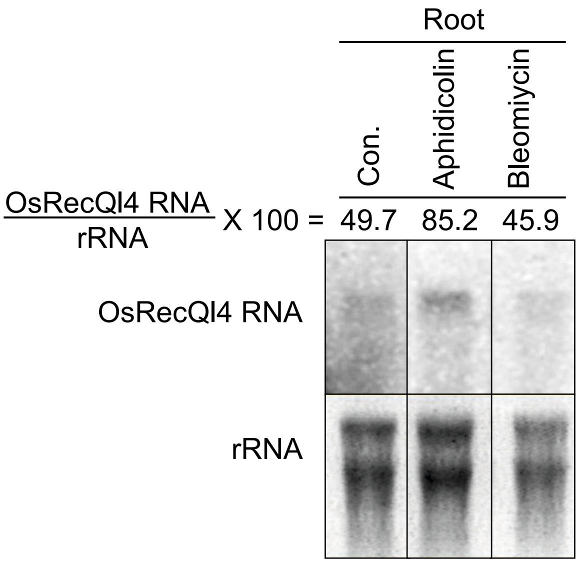

Supplemental Fig. 5. Aphidicolin induces expression of OsRecQl4

Expression of OsRecQl4 in roots increased in response to aphidicolin treatment, but not with bleo mycin treatment. Seedlings were treated with 5mg/L aphidicolin and 5mg/L bleomycin for 7 days. R NA levels were quantified using the image J program (ver.1.43) and relative amounts of OsRecQl4 RNA was calculated using rRNA quantification data.

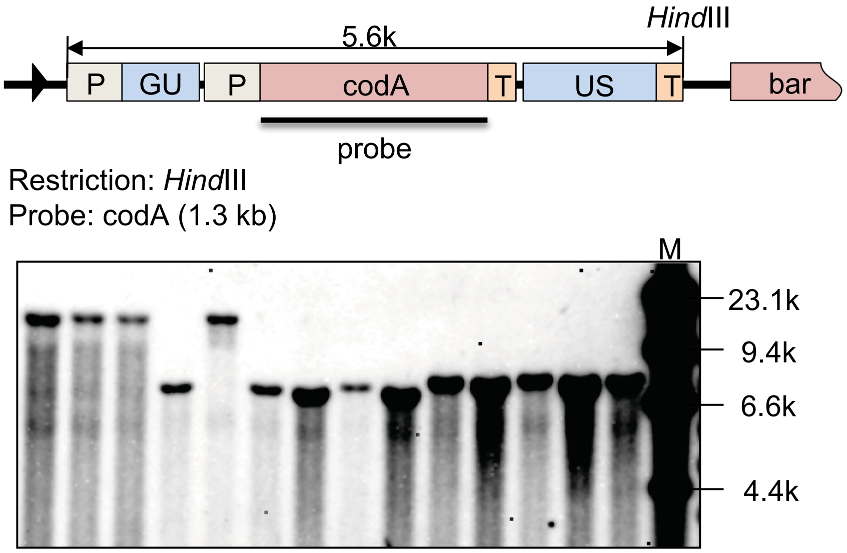

Supplemental Fig. 6. Transgenic calli harboring a single copy of pGU.C.US selected by Southern blotting.

Transformed calli were selected on bialaphos and analyzed by Southern blotting. Independent tra nsgenic callus lines harboring a single copy of the recombination substrate construct were selected and regenerated into plantlets. T0 plantlets were transferred to a greenhouse and grown into matur e plants. Self-fertilized seeds (T1) from each T0 plant were collected separately and used for furthe r analysis

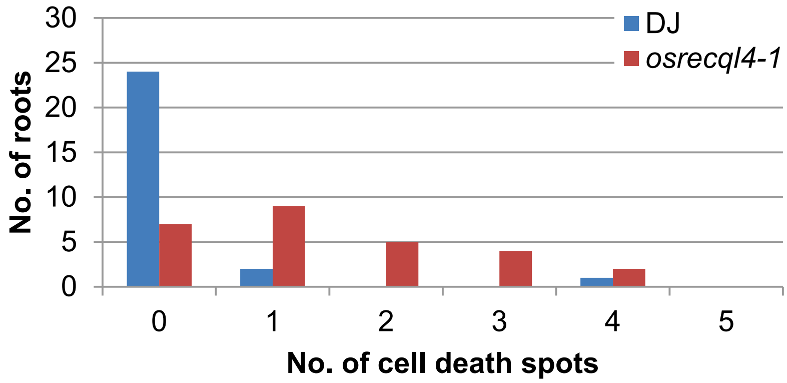

	Average of cell death spots	SD
DJ	0.20	±0.14
osrecql4-1	1.47	±0.18

Supplemental Fig. 7. The osrecql4-1 mutants showed increased numbers of PI stained cells in samples treated with 5mg/L aphidicolin. DJ, wild-type Dongjin; *osrecql4-1*, T-DNA mutant line; Average of cell death spots, number of PI spots per root; SD, standard deviation.

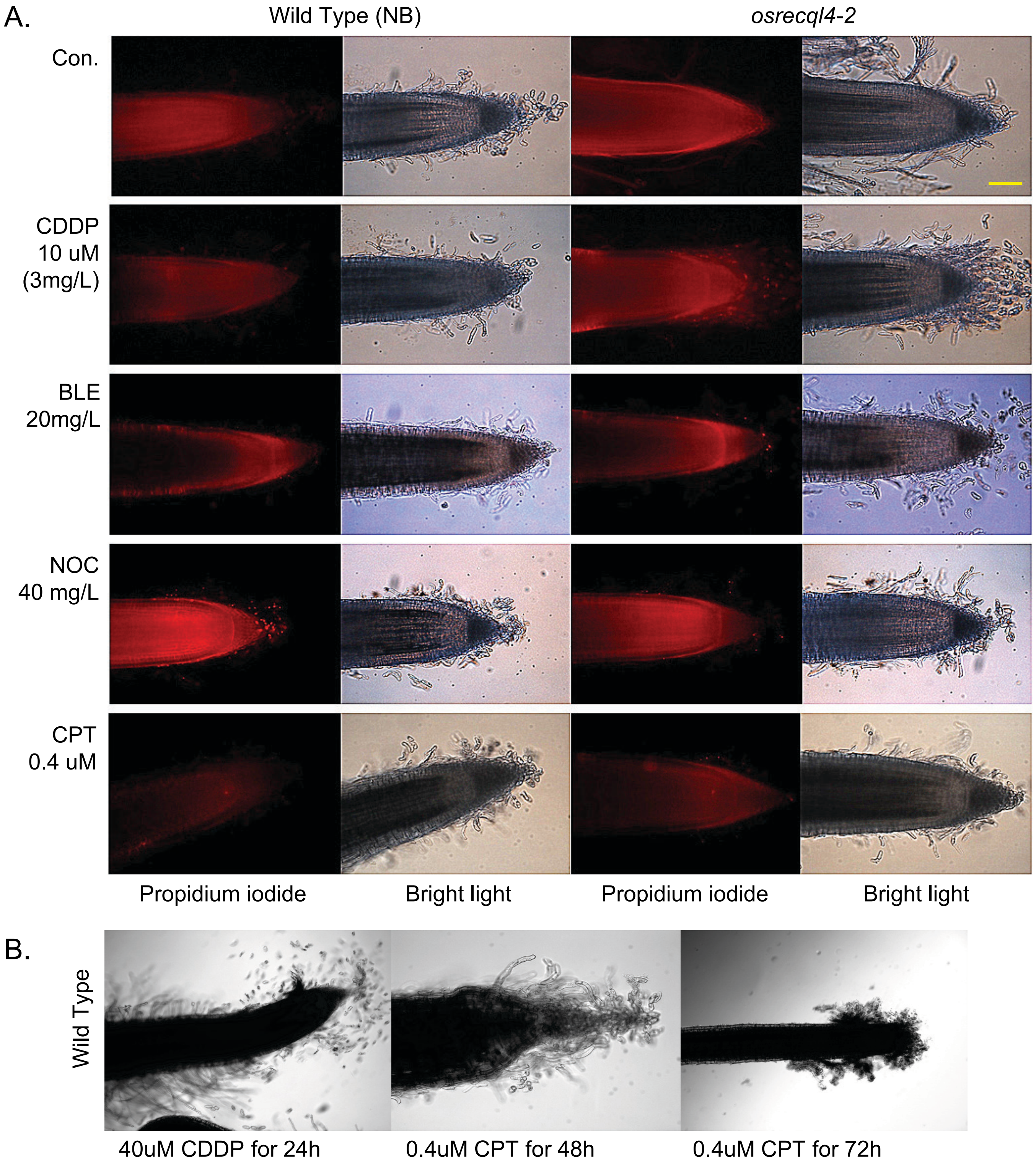

Supplemental Fig. 8.

(A) With other DNA damage inducers [cis-platin (DNA intra-cross link inducer), bleomycin (DSB ind ucer), nocodazole (M-phasearrestor), camptothecin (topoisomerase I inhibitor)] we were unable to observe significant differences between the wild type and osrecql4.

(B) High concentrations or prolonged treatment induced abnormal shapes in the roots and turned t hem opaque. CDDP, cis-platin; BLE, bleomycin; NOC, nocodazole; CPT, camptothecin.
